# Pan-cancer copy number variant analysis identifies optimized size thresholds and co-occurrence models for individualized risk-stratification

**DOI:** 10.21203/rs.3.rs-3443805/v1

**Published:** 2024-01-11

**Authors:** David Raleigh, Minh Nguyen, William Chen, Naomi Zakimi, Kanish Mirchia, Calixto-Hope Lucas

**Affiliations:** University of California San Francisco; University of California San Francisco; UCSF; Univeristy of California San Francisco; Univeristy of California San Francisco; Johns Hopkins University

## Abstract

Chromosome instability leading to accumulation of copy number gains or losses is a hallmark of cancer. Copy number variant (CNV) signatures are increasingly used for clinical risk-stratification, but size thresholds for defining CNVs are variable and the biological or clinical implications of CNV size heterogeneity or co-occurrence patterns are incompletely understood. Here we analyze CNV and clinical data from 565 meningiomas and 9,885 tumors from The Cancer Genome Atlas (TCGA) to develop tumor-and chromosome-specific CNV size-dependent and co-occurrence models for clinical outcomes. Our results reveal prognostic CNVs with optimized size thresholds and co-occurrence patterns that refine risk-stratification across a diversity of human cancers.

Chromosome instability contributes to the genomic complexity of cancer^1^ and is implicated in tumorigenesis, progression, metastasis, and resistance to therapy^2–4^. As a marker of chromosome instability, CNV signatures are increasingly used for clinical risk-stratification of diverse cancer types^5,6^, and pan-cancer databases such as TCGA^7^ have been used to derive prognostic models based on CNVs^6,8^. There is no consensus on the optimal size threshold for defining or reporting CNVs, and CNV co-occurrence patterns that may improve risk-stratification models are incompletely understood.

To test the hypothesis that size-dependent CNV models and co-occurrence patterns may improve clinical risk-stratification, CNV size-dependence was investigated in meningiomas, a tumor that is not represented in TCGA datasets but is associated with recurrent CNVs that can be used for risk-stratification^9,10^. Loss of chromosomes 1p, 6q, and others distinguish biologically aggressive meningiomas^9,10^, but published models have applied inconsistent size thresholds ranging from 5–80% of individual chromosome arms to define meningioma CNVs^9–12^. Using a previously described cohort of 565 meningiomas with long-term clinical outcomes data^11^, we used DNA methylation arrays to define CNVs ranging from individual CpG loci to entire chromosome arms (Extended Data Fig. 1). Next, we used CNVs ranging from 5–95% of each chromosome arm to generate univariate Cox proportional hazards models for postoperative local freedom from recurrence (LFFR) or overall survival (OS). These analyses revealed “size-dependent” CNVs (Fig. 1a), defined as having a maximum area under the curve (AUC) for 5-year LFFR or OS of at least 0.60 that decreased by at least 5% from the maximum AUC as CNV threshold varied (Supplementary Table 1).

The implications of CNV size-dependence for meningioma risk-stratification were investigated using 2 robust models that rely on CNVs to predict postoperative meningioma LFFR. The first, integrated grade, is based on copy number losses of chromosomes 1p, 3p, 4p/q, 6p/q, 10p/q, 14q, 18p/q, and 19p/q at a uniform threshold of 50% of each chromosome arm plus *CDKN2A* loss and mitotic count from histology^9^. The second, integrated score, is based on copy number losses of chromosomes 1p, 6q, and 14q at a uniform threshold of 5% of each chromosome arm plus DNA methylation family^13^ and World Health Organization (WHO) histological grade^10^. We tested each model on our cohort of 565 meningiomas using CNV thresholds ranging from 5–95% (Fig. 1b). Integrated grade reached a maximum AUC for 5-year LFFR of 0.78 at a uniform CNV threshold of 20%, and a maximum AUC for OS of 0.77 at a uniform threshold of 30%. Integrated score reached a maximum AUC for LFFR or OS of 0.76 at a uniform CNV threshold of 5%. The performance of each model degraded with varying CNV size thresholds (Fig. 1b), suggesting that CNV size heterogeneity influences risk-stratification for the most common primary intracranial tumor^14^.

To determine if models based on chromosome-specific CNV size thresholds could improve meningioma risk-stratification, LASSO and elastic net regularized Cox models were trained using optimized CNVs thresholds across the 565 meningiomas in our cohort (Extended Data Fig. 2). Cross-validated AUCs for 5-year LFFR or OS were 0.76 for LASSO models and 0.77–0.78 for elastic net models. CNV size-dependent models identified prognostic chromosome arms that were not included in either integrated grade or integrated score, such as gain of 1q or 17q and loss of 4p, 9p, 10q, or 12q for LFFR, and gain of 1q, 9q, or 10p and loss of 3q, 5p/q, 6p, 9p, 10q, 11p, 13q, 14q, or 18p/q for OS (Fig. 1c), many of which have been previously associated with biologically aggressive meningiomas^11^. There were numerous areas of focal deletion across chromosome arms with size-dependent CNVs that correlated with decreased expression of genes mapping to these loci from RNA sequencing of 502 meningiomas (Fig. 1d, e and Supplementary Table 2). Ontology analysis of genes mapping to focal CNVs revealed dysregulation of metabolic and hormone signaling pathways (Fig. 1f), both of which have been implicated in meningiomas through mechanisms that are poorly understood^15–18^.

Prognostic CNVs from integrated grade, integrated score, and size-dependent LASSO or elastic net models (Fig. 1c) tended to co-occur in individual meningiomas (Fig. 2a). Regularized Cox regression models using co-occurrent CNV pairs identified 1p/22q and 9p/14q co-deletion as important predictors of postoperative LFFR or OS, respectively (Extended Data Fig. 3a). These findings remained significant when accounting for the total number of CNVs per meningioma (“CNV burden”) on multivariate modeling (Supplementary Table 3), and meningiomas with 1p/22q or 9p/14q co-deletion, as defined using optimized CNV size-thresholds, had significantly worse clinical outcomes than meningiomas with these CNVs in isolation of one another (Fig. 2b).

Chromosome 22q loss is a common early alteration in meningiomas^19^, but the prognostic significance of this CNV is limited as subsequent genomic alterations lead to divergent meningioma phenotypes, such as immune infiltration or cell cycle misactivation^11^. Thus, we hypothesized that CNV accumulation in meningiomas may occur sequentially, with some CNVs like loss of chromosome 22q occurring early during tumorigenesis and other CNVs developing later in tumor progression. In support of this hypothesis, hierarchical clustering of meningiomas, binned by CNV burden using optimized size-thresholds, revealed 3 clusters (Fig. 2c, Extended Data Fig. 3b, c). “Early” cluster CNVs, such as loss of 22q, 1p, and 14q, were prevalent regardless of total CNV burden. “Late” cluster CNVs, such as loss of 9p or gain of 1q, were prevalent in samples with higher CNV burden. The third cluster contained uncommon CNVs that did not correlate with total CNV burden. Meningioma CNV burden was associated with worse clinical outcomes, suggesting that progressive destabilization and development of late CNVs is associated with worse prognosis (Extended Data Fig. 3d, e).

To test the broader implications of CNV size thresholds and co-occurrence patterns on cancer risk-stratification, SNP array-derived CNV profiles and clinical outcome data were obtained for 9,885 tumors in TCGA^7^. Nine cancer types, comprising approximately half of TCGA samples analyzed, were identified with CNV size-dependence, which was again defined using prognostic CNV-based models with a maximum AUC for 5-year local PFS or OS of at least 0.60 that decreased by at least 5% from the maximum AUC as CNV threshold varied (Fig. 3a, Supplementary Table 4). There were areas of focal deletion or amplification on size-dependent CNVs across these 9 cancer types, such as gain of 1q and loss of 17q or 21q that were not identified in size-independent cancers (Supplementary Table 5). Ontology analysis of genes mapping to focal CNVs across these 9 cancer types revealed dysregulation of metabolic, developmental, differentiation, biosynthetic, cytoskeletal, and enzymatic pathways (Extended Data Fig. 4).

As in meningioma, size-dependent CNVs for 2 cancer types, glioblastoma (GBM) and cervical squamous cell carcinoma and endocervical adenocarcinoma (CESC), were used as inputs for co-occurrence models (Fig. 3b). In GBM, concurrent 16q loss and 7p gain was associated with worse OS than these CNVs in isolation (Fig. 3c, d). In CESC, concurrent 13q gain and 19p loss, as well as 19p/21q co-deletion, were both significant predictors of OS (Fig. 3c, d). These CNV co-occurrences remained significant predictors for GBM or CESC outcomes in multivariate models that accounted for total CNV burden (Supplementary Table 6). These findings support the clinical relevance of CNV size-dependence and co-occurrence in developing risk-stratification models for human cancer.

In sum, our results demonstrate that CNVs exhibit size-dependence with respect to their prognostic value across multiple cancer types. We find cancer risk-stratification systems using CNVs with chromosome-specific size thresholds and co-occurrence patterns may refine risk-stratification across a diversity of human cancers.

## Methods

### Inclusion and ethics

This study complied with all relevant ethical regulations and was approved by the UCSF Institutional Review Board (13–12587, 17–22324, 17–23196 and 18–24633). As part of routine clinical practice at UCSF, all patients included in this study signed a waiver of informed consent to contribute deidentified data to research.

### Meningioma samples and clinical data

Meningioma samples were collected from two sites, UCSF and Hong Kong University. Samples from the UCSF cohort (n = 200) were selected from the UCSF Brain Tumor Center Biorepository and Pathology Core in 2017, and comprised all available WHO grade 2 and 3 meningioma frozen samples, WHO grade 1 frozen samples with clinical follow-up of greater than 10 years (n = 40) or those with the longest available clinical follow-up less than 10 years (n = 47). The electronic medical record was reviewed for all patients in late 2018, and paper charts were reviewed in early 2019 for patients treated before the advent of the electronic medical record. The Hong Kong University cohort (n = 365) comprised consecutive meningiomas from patients treated at Hong Kong University from 2000 to 2019 with frozen tissue that was sufficient for DNA methylation profiling. The medical record was reviewed for all patients in late 2019. For both cohorts, meningioma recurrence was defined as new radiographic tumor on magnetic resonance imaging after gross total resection, or progression of residual meningioma on magnetic resonance imaging after subtotal resection.

### Meningioma DNA methylation profiling and analysis

DNA methylation profiling was performed as previously described^11^ using the Illumina Methylation EPIC 850k Beadchip (WG-317–1003, Illumina) according to manufacturer instructions. Pre-processing and β-value calculations were performed using the SeSAMe (v1.12.9) pipeline (BioConductor 3.13) with default settings. All DNA methylation profiling was performed at the Molecular Genomics Core at the University of Southern California. Assignment of meningiomas to DNA methylation groups or DNA methylation subgroups was performed using support vector models (https://william-c-chen.shinyapps.io/MeninMethylClassApp/)^11,20^.

### TCGA CNV and clinical outcomes data

TCGA data was collected from the TCGA PanCanAtlas (https://gdc.cancer.gov/about-data/publications/pancanatlas)^21^. Copy number information was obtained using the Copy Number dataset (broad.mit.edu_PANCAN_Genome_Wide_SNP_6_whitelisted.seg). Only primary tumor samples were included by filtering TCGA Biospecimen Core Resource (BCR) barcodes for sample numbers containing the “01” designator. Clinical information was obtained from the TCGA-Clinical Data Resource (CDR) Outcome dataset (TCGA-CDR-SupplementalTableS1.xlsx) and was matched to CNV data by BCR barcode.

### CNV analysis

CNV profiles were generated from DNA methylation data using the SeSaMe package as previously described^11^. The “cnSegmentation” command with default settings and the ‘EPIC.5.normal’ dataset as a copy-number normal control were used.

For both meningioma methylation data and TCGA SNP array data, chromosome segments with mean intensity values less than − 0.1 were defined as lost. Mean intensity values greater than 0.15 were defined as gained. CNV profiling excluded sex chromosomes and p arms of acrocentric chromosomes (13p, 14p, 15p, 21p and 22p). CNV threshold analysis for each CNV profile was performed by measuring the mean intensity value at intervals of 30000 bases along each chromosome arm and summing nonconsecutive gains and losses. The total number of CNV profiles which met each threshold of gain or loss from 5–95% by 5% increments of the chromosome arm were counted. 5-year AUC for meningioma LFFR and OS, and TCGA PFS and OS, were calculated for each threshold using the *survivalROC* package (v1.0.3.1) in R, and the optimal threshold for each CNV was chosen based on the highest AUC for each clinical endpoint. Size-dependent CNVs were defined as those with a maximum 5-year AUC of at least 0.6 with another threshold of less than 95% of that maximum AUC.

CNV network plots were constructed using the *igraph* package (v1.5.1) in R. Plots were constructed using the CNVs selected from regression models, as well as from those identified in the previously published integrated grade^9^ and integrated score^10^ models for meningioma. CNVs were called using their optimal thresholds. In the case of TCGA cancer data, network plots were constructed using size-dependent CNVs and the most important predictors identified in LASSO and Elastic Net Cox regression co-occurrence models. Co-occurrence analysis was limited to pairs of CNVs as sample size was insufficient to analyze the high number of predictors involved when using 3 or more CNVs.

Cluster analysis was performed using CNVs defined with the optimal size-threshold for predicting LFFR. Clustering was done using the *factoextra* (v1.0.7) and *cluster* (v2.1.4) packages in R and visualized with the *ComplexHeatmap* package (v2.15.4).

### Survival analysis and modelling

CNV profiles using the optimal threshold for each CNV were used to train regression models on all available meningioma samples, and for all TCGA samples for size-dependent cancer types (BRCA, CESC, GBM, HNSC, LGG, LUAD, OV, PRAD, and UCEC). LASSO and Elastic net regularized Cox regression models were trained with the concordance index (c-index) for each target endpoint, using the *glmnet* and *cv*.*glmnet* functions from the *glmnet* package (v4.1–8) in R. Elastic net model selection was performed by selecting an optimal alpha value from a range of 0.05 to 0.95 (0.6 for meningioma LFFR, 0.2 for meningioma OS, 0.85 for TCGA PFS, 0.9 for TCGA OS). Model training was performed using 10-fold cross validation. CNV predictors for each model were identified within 1 standard error of the model achieving maximal c-index to reduce over-fitting. A risk metric was calculated for each sample, defined as the product of the regression coefficients and the normalized counts. Model performance was measured with 5-year cross-validation AUC for each model’s respective clinical endpoint using the same training dataset with no hold out validation cohort.

Integrated grade^9^ was assigned to meningioma samples using CNV calls for each threshold, mitoses per 10 high-power fields, and *CDKN2A/B* loss. Integrated score^10^ was assigned using CNV calls for each threshold, WHO grade, and methylation family^13^, the latter which had been previously assigned independently by the authors who developed of this system.

Multivariate Cox proportional hazards analysis was performed using the *survival* package (v3.5–7) in R.

### Focal genomic and ontology analysis

CNV pileup plots demonstrating the proportion of tumors with gains or losses at each position along the chromosome arm were constructed using the *ggplot2* (v3.4.3) package in R. Focal regions of loss were selected by selecting loci along the chromosome arm with a higher proportion of samples demonstrating deletion compared to the surrounding regions. Genes present in regions of interest were identified by cross-referencing positions along the chromosome with the Ensembl (release 109)^22^ database using the *biomaRt* (v2.54.1) package in R.

Meningioma gene expression analysis was performed using RNA-Seq data as previously described^16^. Briefly, RNA sequencing was performed on all 200 of the UCSF samples and 302 of the HKU samples meeting quality metrics. For UCSF samples, library preparation was performed using either the TruSeq RNA Library Prep Kit v2 (RS-122–2001, Ilumina), sequencing was done on an Illumina HiSeq 4000 to a mean of 42 million reads per sample at the UCSF IHG Genomics Core, Quality control of FASTQ files was performed with FASTQC (v0.11.9), and 50 bp single-end reads were mapped to the human reference genome GRCh38 using HISAT2 (v2.1.0) with default parameters. For HKU samples, library preparation was performed using the TruSeq Standard mRNA Kit (20020595, Illumina) and 150 bp paired-end reads were sequenced on an Illumina NovaSeq 6000 to a mean of 100 million reads per sample at MedGenome Inc. Analysis was performed using a pipeline comprised of FastQC for quality control, and Kallisto for reading pseudo alignment and transcript abundance quantification using the default settings (v0.46.2).

Gene ontology and interaction analysis were performed using Cytoscape. In brief, Gene Set Enrichment Analysis (GSEA, v4.3.2) was performed and gene rank scores were calculated using the formula sign(log_2_ fold-change) × −log10(p-value). Pathways were defined using the gene set file Human_GOBP_AllPathways_no_GO_iea_December_01_2022_symbol.gmt, which is maintained by the Bader laboratory. Gene set size was limited to range between 15 and 500, and positive and negative enrichment files were generated using 2000 permutations. The EnrichmentMap App (v3.3.4) in Cytoscape (v3.7.2) was used to visualize the results of pathway analysis. Nodes with FDR q value < 0.05 and p-value < 0.05, and nodes sharing gene overlaps with Jaccard + Overlap Combined (JOC) threshold of 0.375 were connected by blue lines (edges) to generate network maps. Clusters of related pathways were identified and annotated using the AutoAnnotate app (v1.3.5) in Cytoscape that uses a Markov Cluster algorithm to connect pathways by shared keywords in the description of each pathway. The resulting groups of pathways were designated as the consensus pathways in a circle.

### Statistics

All experiments were performed with independent biological replicates and repeated, and statistics were derived from biological replicates. Biological replicates are indicated in each figure panel or figure legend. No statistical methods were used to predetermine sample sizes, but sample sizes in this study are similar or larger to those reported in previous publications. Data distribution was assumed to be normal, but this was not formally tested. Investigators were blinded to conditions during clinical data collection and analysis. Bioinformatic analyses were performed blind to clinical features, outcomes or molecular characteristics. The clinical samples used in this study were retrospective and nonrandomized with no intervention, and all samples were interrogated equally. Thus, controlling for covariates among clinical samples is not relevant. No data points were excluded from the analyses. Statistical analyses were conducted in R (v4.2.2).

## Figures and Tables

**Figure 1:**
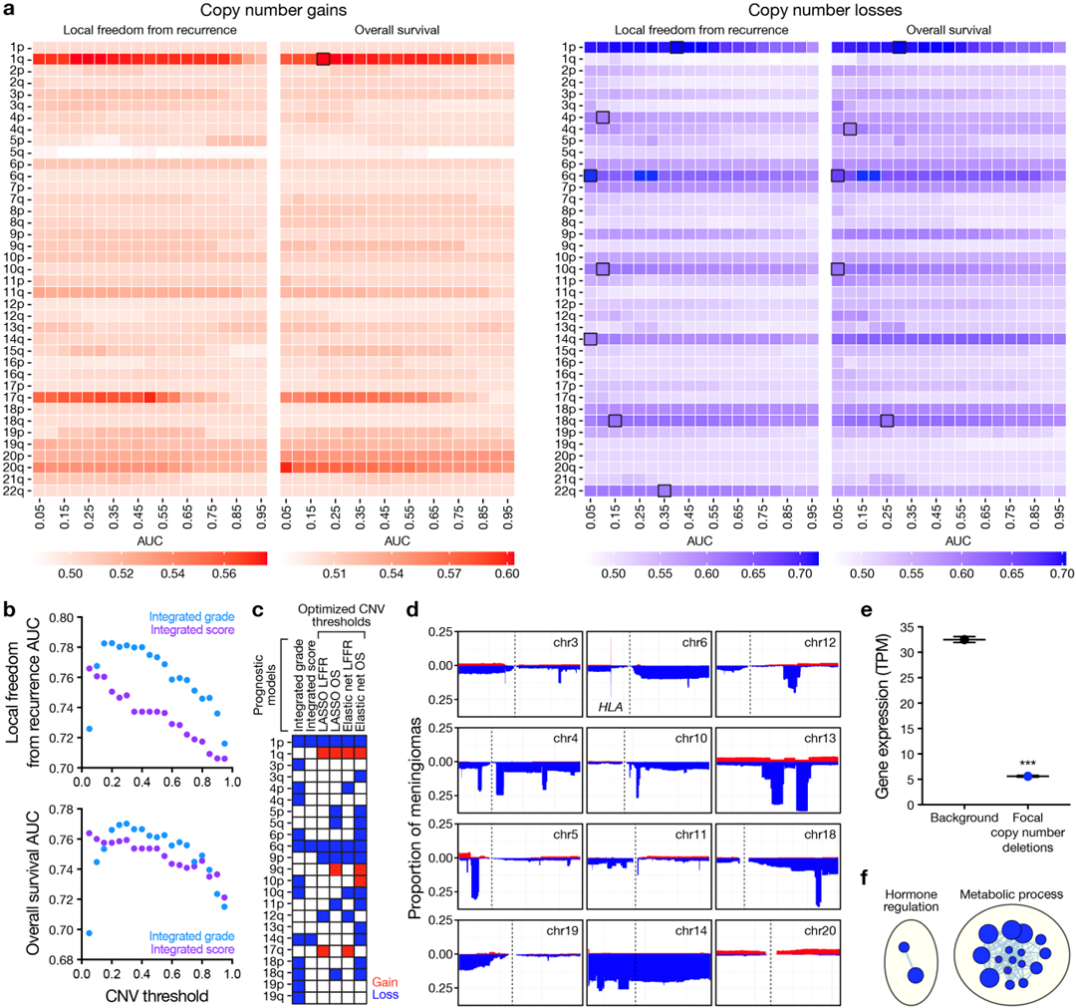
Meningioma risk-stratification models demonstrate CNV size-dependence. **a,** Heatmaps showing area under the curve for univariate Cox models of LFFR or OS based on individual copy number gains (left, red) or individual copy number losses (right, blue). Models were trained using sequential size thresholds requiring ≥5% to ≥95% of chromosome arms to be gained or lost to define CNVs. Boxes show peak AUCs for “size-dependent” CNVs, defined as having a maximum area under the curve (AUC) for 5-year LFFR or OS of at least 0.60 that decreased by at least 5% from the maximum AUC as CNV threshold was varied. n=565 meningiomas. **b**, Previously published meningioma risk-stratification models incorporating CNVs (integrated grade based on histology and a ≥50% CNV threshold, or integrated score based on histology, DNA methylation profiling, and a ≥5% CNV threshold) show decreasing AUC with varying size-thresholds. n=565 meningiomas. **c**, CNVs from previously published meningioma risk-stratification models or from newly-derived size-dependent LASSO or elastic net models for meningioma LFFR or OS. n=565 meningiomas. **d**, CNV profile plots demonstrating focal copy number losses in size-dependent CNVs from LASSO or elastic net models. Chromosomes 14 and 20 are shown as examples of broad/non-focal CNVs. n=565 meningiomas. **e**, Average RNA sequencing expression of genes mapping to regions of focal copy number loss on size-dependent CNVs from LASSO or elastic net models versus genes mapping to other regions on the same chromosomes. n=502 meningiomas. Error bars show standard error of the mean. Student’s t test, p≤0.0001. **f**, Network of gene circuits distinguishing genes mapping to regions of focal copy number loss. Nodes represent pathways and edges represent shared genes between pathways (p≤0.05, FDR≤0.05). n=502 meningiomas.

**Figure 2:**
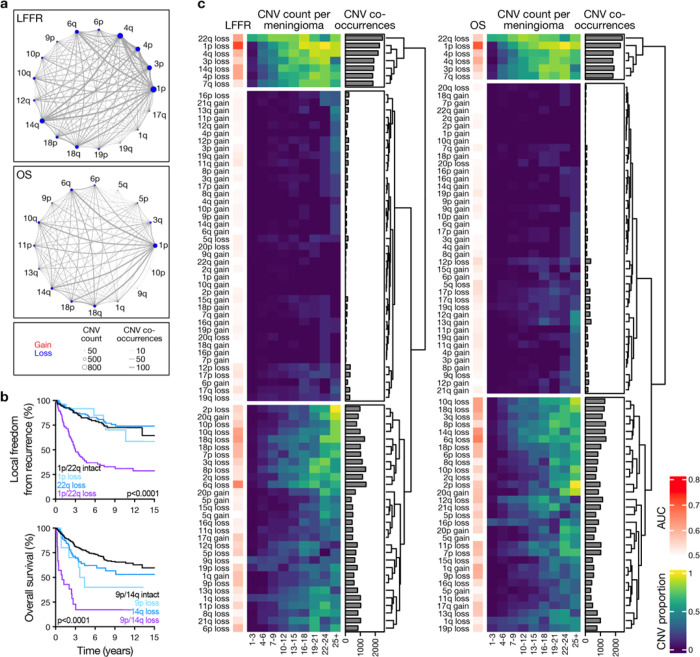
Size-dependent CNV co-occurrence is prognostic for meningioma outcomes. **a**, Network diagrams demonstrating co-occurrence of prognostic size-dependent CNVs from Fig. 1c. **b**, Kaplan-Meier curves comparing meningioma LFFR or OS according to individual CNVs versus co-occurrent CNV pairs identified as the most important predictors of postoperative outcomes in LASSO Cox models from Extended Data Fig. 3a using optimized thresholds for defining CNVs from Fig. 1a. Log-rank tests. n=565 meningiomas. **c**, Heatmap showing unsupervised hierarchical clustering of individual CNVs according to the total number of CNVs per meningioma. CNVs were defined using optimal size thresholds for LFFR or OS models from Fig. 1a.

**Figure 3:**
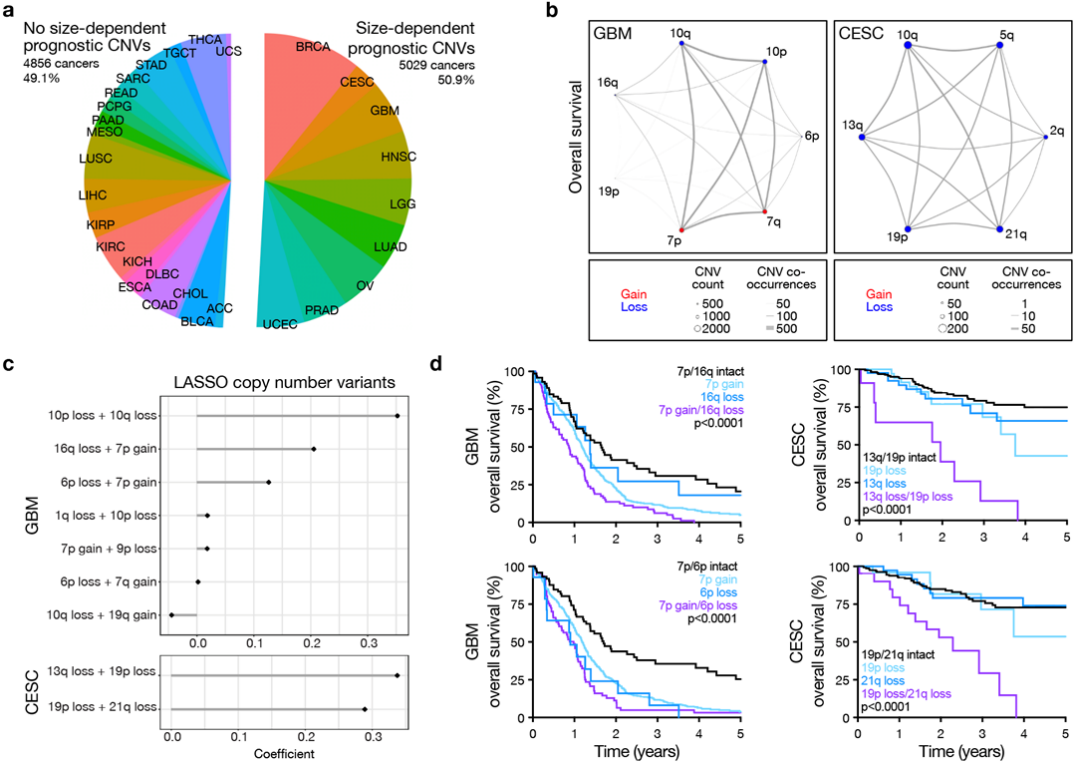
Pan-cancer analyses reveal size-dependent CNV co-occurrence risk-stratification models for half of human cancers. **a**, TCGA SNP-array and clinical outcomes data used in pan-cancer analyses. Cancers with size-dependent prognostic CNVs were defined as having a CNV with a univariate Cox AUC for either PFS or OS of at least 0.60 that dropped by at least 5% from the maximum AUC when varying the size threshold for defining CNVs. **b**, Network diagrams demonstrating co-occurrence of prognostic size-dependent CNVs for GBM or CESC from Supplementary Table 4. **c,** LASSO Cox model coefficients using size-dependent CNV co-occurrence to predict postoperative OS in GBM or CESC. **d,** Kaplan-Meier curves comparing OS for GBM or CESC with individual CNVs versus co-occurrent CNV pairs identified as the most important predictors of postoperative outcomes in LASSO Cox models. Log-rank tests. n= 571 GBM and 294 CESC.

## Data Availability

DNA methylation (n=565) and RNA sequencing (n=502) of the meningiomas analyzed in this manuscript have been deposited in the NCBI Gene Expression Omnibus under the accessions GSE183656 (https://www.ncbi.nlm.nih.gov/geo/query/acc.cgi?acc=GSE183656), GSE101638 (https://www.ncbi.nlm.nih.gov/geo/query/acc.cgi?acc=GSE101638), and GSE212666 (https://www.ncbi.nlm.nih.gov/geo/query/acc.cgi?acc=GSE212666). The publicly available GRCh38 (hg38, https://www.ncbi.nlm.nih.gov/assembly/GCF_000001405.39/), and Kallisto index v10 (https://github.com/pachterlab/kallisto-transcriptome-indices/releases) datasets were used in this study. TCGA data was collected from the publicly available TCGA PanCanAtlas (https://gdc.cancer.gov/about-data/publications/pancanatlas). Copy number information was obtained using the Copy Number dataset (broad.mit.edu_PANCAN_Genome_Wide_SNP_6_whitelisted.seg). Clinical information was obtained from the TCGA-Clinical Data Resource (CDR) Outcome dataset (TCGA-CDR-SupplementalTableS1.xlsx). Source data are provided with this paper.
